# Assessment of the Potential Role of Selected Single Nucleotide Polymorphisms (SNPs) of Genes Related to the Functioning of Regulatory T Cells in the Pathogenesis of Psoriasis

**DOI:** 10.3390/ijms24076061

**Published:** 2023-03-23

**Authors:** Dorota Purzycka-Bohdan, Bogusław Nedoszytko, Marta Sobalska-Kwapis, Monika Zabłotna, Michał A. Żmijewski, Justyna Wierzbicka, Jolanta Gleń, Dominik Strapagiel, Aneta Szczerkowska-Dobosz, Roman J. Nowicki

**Affiliations:** 1 Department of Dermatology, Venereology and Allergology, Medical University of Gdansk, 80-210 Gdansk, Poland; 2 Molecular Laboratory, Invicta Fertility and Reproductive Centre, 81-740 Sopot, Poland; 3 Biobank Laboratory, Department of Oncobiology and Epigenetics, Faculty of Biology and Environmental Protection, University of Lodz, 90-237 Lodz, Poland; 4 Department of Histology, Medical University of Gdansk, 80-211 Gdansk, Poland

**Keywords:** psoriasis, T lymphocytes, regulatory T cells, genetics, single nucleotide polymorphisms

## Abstract

Recent studies have indicated a key role of the impaired suppressive capacity of regulatory T cells (Tregs) in psoriasis (PsO) pathogenesis. However, the genetic background of Treg dysfunctions remains unknown. The aim of this study was to evaluate the association of PsO development with selected single nucleotide polymorphisms (SNPs) of genes in which protein products play a significant role in the regulation of differentiation and function of Tregs. There were three study groups in our research and each consisted of different unrelated patients and controls: 192 PsO patients and 5605 healthy volunteers in the microarray genotyping group, 150 PsO patients and 173 controls in the ARMS–PCR method group, and 6 PsO patients and 6 healthy volunteers in the expression analysis group. The DNA microarrays analysis (283 SNPs of 57 genes) and ARMS–PCR method (8 SNPs in 7 genes) were used to determine the frequency of occurrence of SNPs in selected genes. The mRNA expression of selected genes was determined in skin samples. There were statistically significant differences in the allele frequencies of four SNPs in three genes (*TNF*, *IL12RB2*, and *IL12B)* between early-onset PsO patients and controls. The lowest *p*-value was observed for rs3093662 (*TNF*), and the G allele carriers had a 2.73 times higher risk of developing early-onset PsO. Moreover, the study revealed significant differences in the frequency of SNPs and their influence on PsO development between early- and late-onset PsO. Based on the ARMS–PCR method, the association between some polymorphisms of four genes (*IL4*, *IL10*, *TGFB1*, and *STAT3*) and the risk of developing PsO was noticed. Psoriatic lesions were characterized with a lower mRNA expression of *FOXP3*, *CTLA4*, and *IL2*, and a higher expression of *TNF* and *IL1A* in comparison with unaffected skin. In conclusion, the genetic background associated with properly functioning Tregs seems to play a significant role in PsO pathogenesis and could have diagnostic value.

## 1. Introduction

Psoriasis (PsO) is a chronic, inflammatory, immune-mediated disease with a prevalence of approximately 2–3% in the general population [1]. The development and course of PsO are associated with complex interplay between genetic, environmental, and immunological factors [2]. The activated T lymphocytes as well as cytokines and the growth factors secreted by them constitute a major component of the inflammatory infiltrate within PsO lesions. In particular, dysfunctional helper T cells (Th1, Th17, Th22, and regulatory T cells) are recognized as relevant triggers in PsO development [3].

The regulatory T cells (Tregs) are known as suppressor lymphocytes. Tregs play a fundamental role in the regulation of the immune system and contribute to the prevention of autoimmune diseases by suppressing the immune response [4,5,6]. They are divided into two main groups on the basis of the place of formation, effector mechanisms, and the profile of cytokines produced: (1) primary regulatory lymphocytes, which are formed in the thymus and referred to as nTreg (natural occurring Tregs); and (2) secondary (adaptive), inducible regulatory lymphocytes, referred to as Tr1 and Th3, arising in the periphery from mature T lymphocytes under the influence of antigenic stimulation and/or costimulation (inductive/adaptative iTregs) [7,8]. Tregs inhibit the activity of CD4 and CD8 lymphocytes and thus cause a decrease in the pro-inflammatory cytokine production. The inhibitory effect of Tregs occurs either by direct contact with the target cells (primary suppression) or by secretion of inhibitory cytokines such as IL10 and TGF-β1 (secondary suppression). The quiescence, differentiation, and exhaustion of Tregs are regulated by multilayered factors, including cytokines, antigens, T-cell receptor (TCR) signaling, transcription factors, and epigenetic modifiers [9,10].

Recent studies have revealed that PsO Tregs are characterized by impaired suppressive function leading to an altered T-helper 17/Treg balance [4,11,12,13]. Moreover, it has been shown that Tregs with the phenotype CD4^+^CD25^+high^Foxp3^+^CTLA4^+^ isolated from the peripheral blood and skin of PsO patients have a reduced ability to suppress effector CD4^+^ T cells, contributing to the accelerated proliferation of PsO pathogenic T cells in vivo [14]. However, the mechanism and genetic background of Treg dysfunctions in PsO remain not fully understood [15,16].

The aim of the study was to determine the association of PsO development with selected single nucleotide polymorphisms (SNPs) of genes in which protein products play a significant role in the regulation of the differentiation and function of Tregs.

## 2. Results

### 2.1. Comparison of Variation in Frequency for Tested SNPs of Selected 57 Candidate Genes Related to the Functioning of Tregs Using Microarrays Methods

From 57 selected candidate genes related to the functioning of Tregs (Supplementary Material S1), we selected 283 SNPs and investigated whether these SNPs contribute to PsO risk, including type I (early-onset) and II (late-onset) PsO. The *p*-values reported in our study are not adjusted for multiple testing, although we have included statistical significance values (*p* adj) with the Bonferroni correction in the Tables (Table 1, Table 2 and Table 3). In our study, we did not aim to search for new variants with genetic susceptibility to PsO, but we wanted to check differences in the distribution of SNPs of genes regulating Tregs, which may indirectly affect the risk of developing PsO. However, after taking into account adjustment for multiple testing, we found a strong statistically significant correlation of type I PsO risk with four SNPs: rs3093662 (*p* adj = 1.51 × 10^−6^, OR = 2.73, 95% CI = 1.92–3.88), rs2201584 (*p* adj = 0.005, OR = 1.82, 95% CI = 1.38–2.40), as well as rs3213094 and rs3212220 (*p* adj = 0.020, OR = 0.47, 95% CI = 0.33–0.69) within three different genes (*TNF*, *IL12RB2*, and *IL12B*).

We observed differences in the allele frequencies of 26 SNPs in 14 genes (Table 1). For 11 SNPs (rs3093662, rs2201584, rs12142823, rs10489630, rs6693065, rs4845625, rs2064689, rs10789224, rs7553796, rs4582902, and rs231779), we observed an increased risk of PsO (*p* < 0.005; OR > 1.0). The lowest *p* value was observed for rs3093662 (*TNF*); the G allele carriers had a 2.175 times higher risk of developing PsO. Other SNPs included in Table 1 reduced the risk of PsO (*p* < 0.005; OR < 1.0). All results are presented in the Supplementary Material S2.

Moreover, differences in the frequency of SNPs were noticed between patients with type I PsO, type II PsO and controls.

Out of the 283 polymorphisms analyzed, differences were found in the scope of 25 SNPs in 13 different genes between the group of patients with type I PsO and the control group (Table 2; Supplementary Material S3). In the type I PsO group, incidence of the risk allele in the following six genes was higher than in the control group: *TNF* (rs3093662 G), *IL12RB* (rs2201584 T, rs12142823 T, and rs6693065 G), *CD28* (rs10515944 A), *ENTPD1* (rs4582902 T and rs11188484 A), *IL23R* (rs2064689 A, rs10789224 C, and rs10489630 G), and *IL10RA* (rs2256111 G). There was a tendency observed for these risk allele carriers to develop type I PsO (OR > 1.0). On the other hand, we noticed a decrease in allele frequencies of 14 SNPs (the lowest *p*-value was observed for two SNPs in *IL12B* gene: rs3213094 A and rs3212220 T) among patients with type I PsO compared with the control group, suggesting that their presence had a protective effect against the development of PsO.

Association analysis between the group of patients with type II PsO and the control group revealed different results than those observed in the group of patients with type I PsO (Table 3; Supplementary Material S4).

From the examined SNPs, eight of them were associated with a higher risk of type II PsO (the lowest *p*-values were identified for *IL6R* SNPs: rs7553796, rs4845618, and rs4845625), whereas the protective effect against the development of type II PsO was observed for nine SNPs (the lowest *p*-value was identified for rs8069645 in *STAT3*).

Interestingly, we found the opposite effect of the tested *IL10RA* SNP between results obtained from the analysis of type I PsO and type II PsO. The G allele frequency of rs2228055 (*IL10RA*) was significantly higher in a group of patients with type II PsO compared with the controls, whereas the G allele was significantly less frequent in a group of patients with type I PsO compared with the controls. Thus, the G allele frequency of rs2228055 was observed as a risk factor for developing type II PsO, whereas in the group of type I PsO this allele had a protective effect against PsO (*p* = 0.04, OR = 2.03 and *p* = 0.03, OR = 0.45, respectively) when compared with the control group.

### 2.2. Analysis of the Frequency of Selected Gene Polymorphisms by ARMS–PCR Method

We analyzed eight SNPs in seven genes (*IL4*, *IL6*, *IL10*, *TGFB1*,*CTLA4*, *STAT3*, and *FOXP3*) (Table 4). The CT genotype and the T allele of the −590 *IL4* polymorphism (rs2243250) were observed more often among PsO patients and significantly increased the risk of PsO (OR = 2.52, *p* = 0.0001; OR = 2.0, *p* = 0.0002). The CC genotype and the C allele of the −590 *IL4* were significantly less frequent in patients than in the control group and their presence decreased the risk of PsO (OR = 0.38, *p* < 0.001 and OR = 0.5, *p* = 0.002, respectively). The protective effect against PsO development was also observed for the GG genotype and the G allele of the −1082 *IL10* (rs1800896) (OR = 0.56, *p* = 0.04; OR = 0.7, *p* = 0.02). Both the TT genotype and the T allele of the *TGFB1* codon10 polymorphism (rs1982073) were significantly more frequent in the group of patients (*p* < 0.0001), and their presence increased the risk of PsO more than five times (OR = 5.5) in the case of the TT genotype and more than four times (OR = 4.47) in the case of the T allele. The TC genotype, CC, and the C allele were significantly rarer in the PsO group compared with the controls, and their presence reduced the risk of PsO (OR = 0.3, OR = 0.24, and OR = 0.22, respectively). Additionally, the presence of the CC genotype of the *STAT3* polymorphism (rs2293152) was found to exert a protective effect against PsO development (OR = 0.31; *p* = 0.04). In the case of other polymorphisms, no statistically significant differences were noticed.

### 2.3. Analysis of Expression of Selected Genes in Skin Biopsies of PsO Patients and Controls

**Figure 1:**
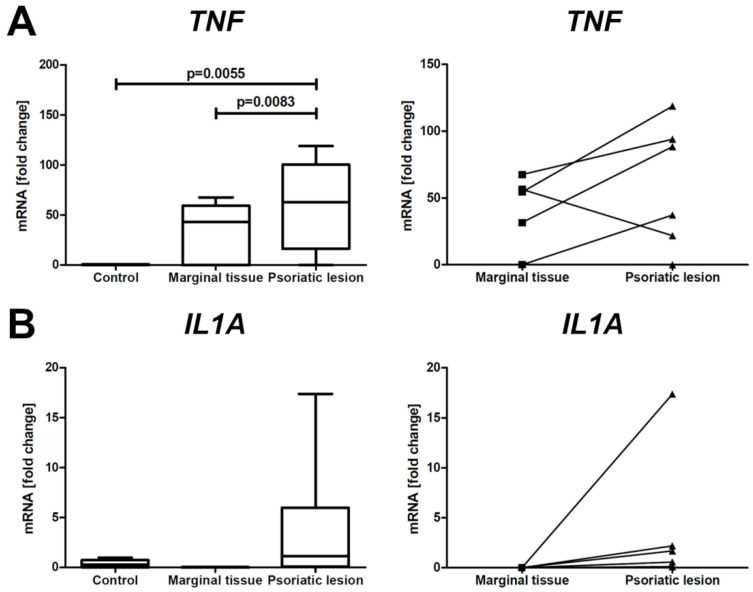
mRNA expression of pro-inflammatory genes (*TNF* and *IL1A*) in healthy skin, marginal tissue, and PsO lesions. The graphs on the left compare the expression levels of *TNF* (**A**) and *IL1A* (**B**) in PsO lesions and their marginal tissue (n = 6) with the skin of healthy individuals (n = 6). The graphs on the right show the paired gene expressions of *TNF* (**A**) and *IL1A* (**B**) in PsO skin lesions and marginal tissue from each individual patient (n = 6).

Within the PsO plaques, a significantly higher mRNA expression of *TNF* (Figure 1A) was observed in comparison with the skin surrounding the lesions and the skin of healthy individuals, whereas, in spite of the observed tendency, the changes in the expression of *IL1A* were not statistically significant between the groups (Figure 1B).

**Figure 2:**
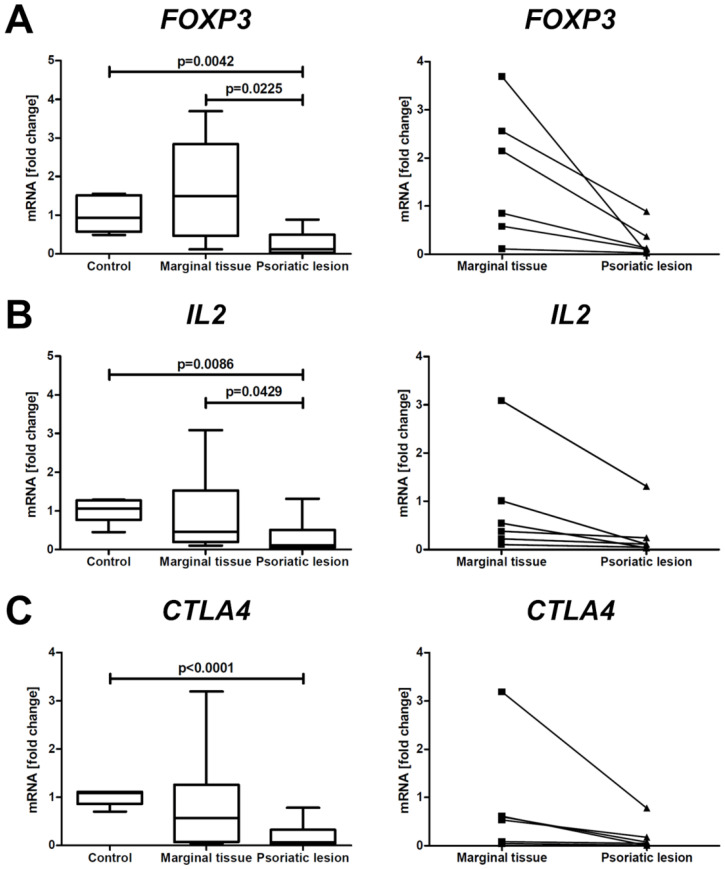
mRNA expression of anti-inflammatory genes (*FOXP3*, *IL2*, and *CTLA4*) in healthy skin, marginal tissue, and PsO lesions. The graphs on the left compare the expression levels of *FOXP3* (**A**), *IL2* (**B**), and *CTLA4* (**C**) in PsO lesions and their marginal tissue (n = 6) with skin of healthy individuals (n = 6). The graphs on the right show the paired gene expressions of *FOXP3* (**A**), *IL2* (**B**), and *CTLA4* (**C**) in PsO skin lesions and marginal tissue from each individual patient (n = 6).

Compared with healthy skin and marginal tissue, PsO lesions showed a significant reduction in the mRNA expression of the *FOXP3* (Figure 2A), *IL2* (Figure 2B), and *CTLA4* (Figure 2C) genes in which protein products are associated with Tregs development and activation.

## 3. Discussion

The Tregs play a crucial role in the inhibition of the excessive immune response and mediate in the maintenance of immunological homeostasis. It has been shown that PsO patients have functional defects in CD4^+^CD25^+^Foxp3^+^ Tregs [17]. Disturbances in Treg activity as well as factors contributing to increased Treg inhibition and/or impaired stimulation appear to be involved in the etiology of PsO [17,18]. Our results indicate the possible role of the genetic background associated with defects of Tregs in the PsO pathogenesis. After adjustment for multiple testing, the study revealed significant differences in the allele frequencies of four SNPs in three genes, including *TNF*, *IL12RB2*, and *IL12B*, between type I PsO and controls. However, there are likely differences between the allele frequencies for another 22 SNPs in 12 genes *(IL13/TH2LCRR*, *IL12RB2*, *IL23R*, *IL6R*, *RUNX1*, *NRP1*, *IL17RA*, *PPARG*, *ENTPD1*, *CTLA4*, *STAT5B*, and *CD274*) in the group of all PsO patients compared with the control group. The products of these genes include cytokines and their receptors, antigens, and transcription factors. All of them are involved in the production, differentiation, and activity of Tregs, and that is why they are worth discussing.

The tumor necrosis factor (TNF) is a pro-inflammatory cytokine produced by monocytes and macrophages as well as other immune cells, including dendritic cells, B cells, activated natural killer cells, and T cells [19]. The TNF plays a significant role in the pathogenesis of various inflammatory and autoimmune diseases. It is believed that this cytokine may affect Treg activity [20]. Tregs have been suggested to suppress Th1 and Th17 effector T cell responses. Some studies have reported that TNF reduces the suppressive function of Tregs mainly by decreasing FOXP3 expression and therefore contributes to chronic inflammation [21]. However, other studies have shown that TNF enhances the suppressive function of Tregs [22]. The interactions between TNF and Tregs have not been clearly defined. A study investigating rheumatoid arthritis found that memory Tregs (CD4^+^CD45RO^+^CD25^+^CD127^low^) can acquire the ability to produce pro-inflammatory cytokines, including not only IFN-γ and IL17, but also TNF, in response to inflammatory stimuli [23]. It is postulated that genetic factors associated with the functioning of TNF may play a role in the pathogenesis of PsO. It has been found that *TNF* (SNP rs3093662) is a susceptible gene for PsO in the Chinese population [24,25]. Our research also indicated a potential role of rs3093662 (*TNF*) in PsO etiology as we noticed that G allele carriers had a 2.175 times higher risk of developing PsO (*p* = 2.40 × 10^−6^). Similarly to other studies, we observed a significant increase in mRNA *TNF* in PsO [26,27]. Therefore, it seems likely that the involvement of genetic factors in TNF function may indirectly contribute to the Treg-mediated response in PsO, but this requires further research.

The role of *IL12B* in the genetics of PsO was explored broadly, and potentially causative variants of *IL12B* were identified throughout genome-wide association studies (GWAS) worldwide [28,29,30,31,32,33]. *IL12B* encodes the IL-12p40 subunit of two distinct heterodimeric cytokines, IL12 and IL23, which are involved in the immune responses. SNPs in *IL12B*- and *IL12B*-related genes including *IL12A*, *IL23A* (encoding the IL-12p35 and IL-23p19 subunits, respectively), and genes encoding the receptors of IL12 and IL23 (*IL12RB1*, *IL12RB2*, and *IL23R*) are suggested to play a role in PsO susceptibility [29]. Hunter et al. highlighted the unique significance of these two cytokines as they drive the development of distinct subsets of T-cells [34]. IL-12 contributes to the induction and enhancement of Th1 cells, whereas IL-23 is associated with the generation of Th17 response and the production of IL17 [35]. Through subunits of its receptor, IL-12 activates a cascade of signaling factors including STAT4 and NFK𝛽, and thus promotes the production of pro-inflammatory Th1-type cytokines such as TNF𝛼 and IFN-𝛾 [36]. Moreover, *IL12* induces IFN-γ expression by Tregs and inhibits Treg proliferation and Foxp3 expression [37]. In this study, we confirmed that SNPs rs3213094 and rs3212220 in *IL12B*- and *IL12B*-related genes (rs2201584 in *IL12RB2*) were associated with PsO pathogenesis. Our results are parallel with earlier studies which have reported that rs3212220 confers protection against PsO [29,32].

Literature data indicate the concept of two subtypes of PsO based on the age of onset, showing not only different epidemiological and immunocytochemical features but also genetic ones [38,39,40]. Our study revealed significant differences in the frequency of SNPs and their influence on PsO development between early- and late-onset PsO, thus emphasizing various genetic backgrounds of the above types of PsO. The greatest differences in this respect concerned the polymorphism of the *IL10RA* gene. IL10 receptor (IL10R) signaling in Tregs has been implicated in the downregulation of Th17 cell-mediated inflammation [41], but the underlying molecular mechanisms and functional relevance of this process remain unclear. In one study, abolition of IL10Ra signaling in Tregs was shown to increase the activation of dendritic cells and the production of Th17-inducing cytokines [42]. Interestingly, we found the opposite effect of tested *IL10RA* SNP (rs2228055) when comparing obtained results from the analysis of type I PsO and type II PsO, which requires further evaluation. Moreover, we noticed the protective effect against PsO development for the GG genotype and the G allele of the −1082 *IL10* polymorphism (rs1800896). In the literature, data concerning the possible role of rs1800896 are controversial [43]. Some authors highlighted its significance in predisposition to PsO [44,45,46,47]. However, in contrast with these results, Reich et al. [48], Baran et al. [49], and Peddle et al. [50] found no significant role of rs1800896 polymorphism in PsO patients.

The IL4 is an anti-inflammatory cytokine which affects the function of many types of cells. It inhibits Th1/Th17 cells and decreases the cytokine level of IFN-γ [51]. Tregs exposed to IL4 are more likely to suppress the proliferation of naïve CD4^+^ T cells as well as to inhibit IFN-γ production by CD4^+^ T cells. Some authors concluded that the anti-inflammatory properties of IL4 could be partly mediated by the effect on the function of Tregs [52]. Yang WC et al. confirmed the essential role of IL4 in supporting Treg-mediated immune suppression [53]. Another study revealed a decreased serum level of IL4 and *IL4* gene expression in PsO patients [54]. It has been suggested that the *IL4* polymorphism (rs2243250) might be associated with genetic susceptibility to autoimmune diseases [55]. Indhumathi S et al. postulated that this polymorphism is protective against PsO [56]. Our study also revealed a potential role of *IL4* polymorphism (rs2243250) in PsO development. We noticed that the presence of the CC genotype and C allele of rs2243250 had a protective effect against the development of PsO. However, we have also shown a significant association between the CT genotype and T allele of rs2243250 and an increased risk of PsO of 2.5-fold and 2-fold, respectively.

Many cytokines seem to be involved in the pathogenesis of PsO, including transforming growth factor beta 1 (TGF-β1), whose function is not limited to the inhibition of keratinocyte proliferation [57]. TGF-β1 signaling is essential for the differentiation of both thymus-derived nTregs and peripherally-induced iTregs [58]. Divyapriya et al. revealed a significantly decreased serum level of TGF-β1 in PsO patients compared with controls [54]. The research performed among the population of Iraq showed a significant association between *TGFB1* gene polymorphism at codon 10 (T869C, rs1982073) and 25 (G915C, rs1800471) with PsO susceptibility and revealed a significantly lower mean serum TGF-β1 level in PsO patients compared with controls [59]. El-Hadidi et al. reported an association between *TGFB1* gene polymorphism at codon 10 (rs1982073) with susceptibility to PsO in Egyptian patients [60]. To our knowledge, the presented study is the first evaluating the significance of *TGFB1* codon 10 polymorphism (rs1982073) in a Caucasian PsO population. We found that both the TT genotype and the T allele of the *TGFB1* codon 10 polymorphism (rs1982073) were significantly more frequent in the group of patients, and their presence increased the risk of PsO more than five times in the case of the TT genotype and more than four times in the case of the T allele. The TC genotype, CC, and the C allele were significantly rarer in the PsO group compared with controls and their presence decreased the risk of PsO (OR = 0.3, OR = 0.24, and OR = 0.22, respectively).

The forkhead box P3 (FOXP3) is a key transcription factor in the development and function of Tregs [17]. Previous studies have demonstrated a genetic association between the *FOXP3* gene and PsO [61,62]. *FOXP3* gene polymorphism may change FOXP3 protein activity, leading to the dysfunction of Tregs. The *FOXP3* polymorphisms, including rs2232365, appear to contribute to the risk of PsO among the Chinese majority Han population [61]. In our study, we did not observe a significant impact of rs2232365 on the risk of PsO development. However, we found decreased mRNA *FOXP3* levels within PsO lesions compared with both control (healthy) and marginal tissue. These observations are in accordance with other studies. Kuang et al. revealed that the number of Tregs and the level of *FOXP3* mRNA were lower in the spleen of mice with PsO than in the control group (*p* < 0.05) [63]. Furthermore, the authors reported the association of clinical improvement after methotrexate treatment with an increase in the number of Tregs and the Foxp3 mRNA level in PsO dermatitis in mice [63].

According to the literature data, IL2 is necessary for the growth and survival of Tregs in the peripheral lymphatic tissues [64]. Moreover, the relative deficiency of IL2 observed in the course of some autoimmune disorders contributes to the disturbance of Treg homeostasis, which reinforces the vicious circle of tolerance violation and the development of chronic inflammation [65]. Wang et al. reported that psoriatic arthritis (PsA) patients exhibited peripheral low Treg numbers [66]. However, the combination treatment with low-dose IL2 restored this number and reduced PsA activity without noticeable side effects [66]. Our study revealed a significant decrease in the mRNA expression of the *IL2* gene in PsO lesions compared with the skin of healthy volunteers.

Gene expression studies of the skin showed a significant decrease in the mRNA expression of genes in which protein products are essential for proper suppressor activity of Tregs. Our research showed that PsO lesions were characterized not only with lower mRNA levels of *FOXP3* and *IL2* but also with lower mRNA levels of the cytotoxic T lymphocyte-associated antigen 4 (*CTLA4*) gene. CTLA4 is a critical negative regulator of immunological responses. CTLA4 plays an important role in the process of the proper functioning of Tregs [67]. Loss of CTLA4 in Tregs contributes to impaired Treg suppressive activity and the abnormal function of conventional T cells [68]. Moreover, we noticed that the mRNA gene levels of proinflammatory cytokines, such as *TNF* and *IL1A*, were more increased within PsO plaques than within unaffected skin. Because IL1 signaling leads to the release of many proinflammatory cytokines, including TNF*α*, IL17A, and IL6, it has been implicated in preventing immune suppression by Tregs [69]. Therefore, it is believed that IL1 and its associated signaling pathways may contribute to the functional deficiency of Tregs in PsO [69].

### Limitations of the Study

The limitation of the presented study is the size of the tested group. Larger population-based studies are needed to confirm our findings as well as to assess the possible relationship between gene SNPs and disease severity. The study of possible interactions between the allele and/or genotype of the tested gene SNPs could be also helpful in further understanding the genetic basis of PsO. Furthermore, we have not tested the impact of PsO therapies on Tregs. It was suggested that many current or prospective treatments for PsO appear to affect Treg frequency and/or functionality and the Th17/Treg balance in PsO [4].

## 4. Materials and Methods

### 4.1. PsO Group

The PsO group included 192 adult (age > 18 years), unrelated patients (men and women) with PsO vulgaris who were admitted to the Department of Dermatology as well as to the Dermatology Outpatient Clinic of the Medical University of Gdansk. The diagnosis was confirmed by histology or clinical examination. The recruited patients had not been treated systemically for PsO (e.g., cyclosporine, methotrexate, retinoids, or photochemotherapy) for at least the previous 3 months and had not received topical anti-psoriatic treatment for the previous 3 weeks. Patients suffering from other chronic skin disorders, systemic inflammatory diseases, or malignancies as well as those treated with biological drugs were excluded. All patients were exclusively of Polish descent.

The patients were divided into two subgroups: type I PsO (early-onset PsO; onset age < 40 years; n = 147) and type II PsO (late-onset PsO; onset age ≥ 40 years; n = 45) [39].

### 4.2. Control Group

A total of 5605 adult (age > 18 years) donors (men and women) were enrolled in this study with their genetic data. The control group (Caucasian population, Central Europe) were recruited between 2010 and 2012 as a part of the TESTOPLEK research project and registered as the POPULOUS collection at the Biobank Lab of The Department of Molecular Biophysics of The University of Łódź, Poland [70]. The exclusion criteria involved PsO, family history of PsO, current or past history of malignancy (including myeloid disorders), or bone marrow transplantation.

Peripheral blood was collected from all PsO patients, whereas saliva (for microarray genotyping) and blood samples (for ARMS–PCR genotyping) were collected from donors from the control group.

### 4.3. DNA Isolation

For the samples from the POPULOUS collection, procedures concerning DNA extraction and sample processing have previously been described [71,72]. Briefly, saliva samples first underwent lysis and protein precipitation followed by DNA precipitation with isopropanol. A total of 50 uL of water (molecular grade) was used for the rehydration of precipitated DNA.

For the group of PsO patients, genomic DNA was extracted from whole blood using the MagNa Pure LC 2.0 (Roche, Basel, Switzerland).

In both cases, the NanoDrop 1000 (Thermo Fisher Scientific Inc., Waltham, MA, USA) and Qubit 2.0 (Thermo Fisher Scientific Inc., Waltham, MA, USA) were used to determine DNA quality and concentrations. Afterward, DNA samples underwent a PCR reaction for sex verification. The same method was performed as in our previous study [73].

### 4.4. Microarrays Analysis

As in our previous study [61], the DNA samples were genotyped using Infinium CoreExome microarrays (Illumina Inc., San Diego, CA, USA). Array genotyping was performed according to standard Illumina protocols. Microarray scan data were then converted to genotypes using Genome Studio (Illumina Inc., San Diego, CA, USA). SNPs were limited to the autosomes and a 10% GenCall > 0.4. We further filtered the dataset on sample call rates, and only those >0.94 were included. The results were then exported from GenomeStudio using PLINK Input Report Plug-in v2.1.4 by forward strand.

### 4.5. ARMS–PCR

Genomic DNA was prepared from blood samples of 150 randomly selected PsO patients and 173 randomly selected controls using Blood DNA Prep Plus in accordance with the protocol of the manufacturer (A&A Biotechnology, Gdynia, Poland). The selected gene polymorphisms (rs2243250, rs1800795, rs1800896, rs1982073, rs5742909, rs2293152, rs4796793, and rs2232365) were analyzed using the amplification refractory mutation system–PCR (ARMS–PCR) method, using specific sequences of oligonucleotides.

### 4.6. mRNA Level of the Genes in Skin Samples

For mRNA isolation, 4 mm diameter biopsies were taken from disease-affected skin (PsO lesions) of the buttocks and additionally from potentially unaffected margins (marginal tissue in the distance of about 2 cm from the plaque) of 6 volunteers from the PsO group and from healthy skin of the buttocks of 6 volunteers from the control group. The primer sequences used in the study are given in Table 5.

Briefly, the relative mRNA levels for the genes analyzed (*TNF*, *IL1A*, *FOXP3*, *IL2*, and *CTLA4*) were established using the quantitative polymerase chain reaction (qPCR) in matching samples (PsO lesion and marginal skin) and control biopsies. Following mechanical homogenization of tissue samples, total RNA was isolated using the Total RNA Mini kit (A&A Biotechnology, Gdynia, Poland). The amount and purity of the RNA samples were determined spectrophotometrically (Epoch BioTek, Winooski, VT, USA). One microgram of RNA was used for reverse transcription performed with a RevertAid™ First Strand cDNA Synthesis Kit (Thermo Scientific, Waltham, MA, USA). The real-time PCR reaction was carried out using the StepOnePlus™ Real-Time PCR System (Life Technologies-Applied Biosystems, Grand Island, NY, USA) with a SensiFAST SYBR^®^ No-ROX PCR kit (Bioline, London, UK) according to the manufacturer’s protocol. The expression of genes was normalized using the comparative ΔΔ-*C*_t_ method, using *RPL37* as a housekeeping gene, followed by calibration (fold change) to normalized expression data of samples from control patients (ratio = 1).

### 4.7. Statistical Methods

#### 4.7.1. Microarray Data Set

We extracted 57 candidate gene regions (Supplementary Material S1) related to the functioning of regulatory T lymphocytes from the entire dataset, which resulted in the selection of 833 SNPs. Selected genetic data were filtered and only variants with minor allele frequencies above 5% were included. Subsequently, the Hardy–Weinberg equilibrium was also tested and SNPs with a *p*-value below 0.05 were excluded from the association analysis. A total of 283 SNPs remained for statistical analyses.

After the quality of all data was confirmed for accuracy, case—control association analysis was performed using the PLINK toolset (ver. 1.07) [74].

Differences in allele frequencies between PsO patients and population controls were tested for each SNP using a 1-degree-of-freedom Chi-square test. The allelic odds ratios were calculated with a confidence interval of 95%. To reduce experiment error rates, the adjustment for multiple testing using the Bonferroni correction was applied.

#### 4.7.2. ARMS–PCR Data Set

Statistical calculations were performed using the Statistica 12.0 software package (StatSoft, Inc., Tulsa, OK, USA, 2015). The χ^2^ analysis was used to compare the observed number of genotypes with that expected for a population in a Hardy–Weinberg equilibrium as well as to examine the significance of the differences in the observed genotypes and alleles between study groups. A logistic regression model was applied to calculate the odds ratio (OR) and the 95% confidence interval (CI). A *p*-value < 0.05 was considered to be statistically significant.

#### 4.7.3. mRNA Gene Expression Data Set

The data are presented as median values with range (min–max) and were analyzed with a Student’s *t*-test (for two groups) or a one-way analysis of variance with appropriate post-hoc tests (for more than two groups). All statistical analyses were performed using GraphPad Prism 7.00 (GraphPad Software, San Diego, CA, USA). A *p*-value < 0.05 was considered to be statistically significant.

## 5. Conclusions

In summary, we report that the tested SNPs correlated with the risk of PsO by influencing the production of cytokines, antigens, growth factors, and cells that participate in the immune response together with Tregs.

Further studies on the impact of genetic factors on the production, differentiation, and functioning of Tregs in PsO are required.

## Figures and Tables

**Table 1:** Comparison of the allele frequency of SNPs in PsO patients and controls.

CHR	SNP	A1	F_A	F_U	CHISQ	*p*	*p* adj	OR	SE	L95	U95	Overlapped Gene	Annotation
6	rs3093662	G	0.1094	0.05343	22.25	**2.40 × 10^−6^**	0.0007031	**2.175**	0.1688	1.563	3.029	*TNF*	intronic
5	rs1295686	A	0.1719	0.2494	12.01	**0.0005296**	0.1552	**0.6246**	0.137	0.4775	0.817	*IL13/TH2LCRR*	intronic, non-coding intronic
5	rs20541	T	0.1719	0.2475	11.48	**0.0007035**	0.2061	**0.631**	0.137	0.4824	0.8254	*IL13/TH2LCRR*	coding nonsyn, non-coding
1	rs2201584	T	0.1953	0.1399	9.381	**0.002192**	0.6422	**1.493**	0.1316	1.153	1.932	*IL12RB2*	intronic
5	rs3213094	A	0.1354	0.198	9.24	**0.002367**	0.6936	**0.6343**	0.151	0.4718	0.8527	*IL12B*	intronic
5	rs3212220	T	0.1354	0.198	9.24	**0.002367**	0.6936	**0.6343**	0.151	0.4718	0.8527	*IL12B*	intronic
1	rs12142823	T	0.1731	0.1238	7.819	**0.005171**	1	**1.482**	0.1415	1.123	1.955	*IL12RB2*	5utr, 5upstream
1	rs10489630	G	0.4453	0.3795	6.808	**0.009076**	1	**1.312**	0.1045	1.069	1.611	*IL23R*	intronic
1	rs4845625	T	0.5547	0.4908	6.061	**0.01382**	1	**1.292**	0.1044	1.053	1.586	*IL6R*	intronic, non-coding intronic
1	rs2064689	A	0.3411	0.2869	5.323	**0.02104**	1	**1.287**	0.1096	1.038	1.596	*IL23R*	intronic
1	rs10789224	C	0.3385	0.2847	5.264	**0.02177**	1	**1.286**	0.1099	1.037	1.595	*IL23R*	intronic
21	rs2268288	C	0.1623	0.2099	5.076	**0.02426**	1	**0.7292**	0.1407	0.5535	0.9608	*RUNX1*	intronic, 5upstream
1	rs6693065	G	0.25	0.203	5.045	**0.0247**	1	**1.309**	0.1202	1.034	1.656	*IL12RB2*	intronic
10	rs734187	T	0.237	0.2893	4.968	**0.02583**	1	**0.7628**	0.1218	0.6008	0.9685	*NRP1*	intronic, 3downstream
22	rs2241042	A	0.3455	0.4017	4.862	**0.02746**	1	**0.7863**	0.1093	0.6346	0.9741	*IL17RA*	intronic, non-coding intronic
3	rs4135280	C	0.03646	0.06396	4.754	**0.02923**	1	**0.5537**	0.275	0.323	0.9493	*PPARG*	intronic, 3downstream
10	rs1044268	A	0.1328	0.1753	4.655	**0.03096**	1	**0.7208**	0.1524	0.5346	0.9717	*NRP1*	3utr
21	rs2014300	A	0.1276	0.1682	4.398	**0.03598**	1	**0.7234**	0.155	0.5339	0.9803	*RUNX1*	intronic
1	rs7553796	A	0.5312	0.477	4.383	**0.0363**	1	**1.243**	0.104	1.014	1.524	*IL6R*	intronic
1	rs4537545	T	0.3125	0.364	4.258	**0.03905**	1	**0.7943**	0.1118	0.6379	0.9889	*IL6R*	intronic, non-coding intronic
10	rs4582902	T	0.5262	0.4726	4.254	**0.03915**	1	**1.239**	0.1042	1.01	1.52	*ENTPD1*	intronic, non-coding intronic
2	rs231779	T	0.4583	0.4074	3.989	**0.0458**	1	**1.231**	0.1042	1.004	1.51	*CTLA4*	intronic, 5upstream, non-coding intronic
17	rs9900213	T	0.1094	0.1458	3.975	**0.04617**	1	**0.7197**	0.1657	0.5201	0.9958	*STAT5B*	intronic, non-coding intronic
9	rs10481593	A	0.1875	0.231	3.972	**0.04627**	1	**0.7683**	0.1327	0.5924	0.9964	*CD274*	intronic, non-coding intronic
10	rs734186	T	0.3698	0.4206	3.936	**0.04726**	1	**0.8083**	0.1074	0.6549	0.9978	*NRP1*	intronic, 3downstream
1	rs4129267	T	0.3073	0.3564	3.904	**0.04816**	1	**0.8013**	0.1124	0.6429	0.9986	*IL6R*	intronic, 5upstream, non-coding intronic

CHR—chromosome; SNP—single nucleotide polymorphism; A1—minor allele name (based on whole sample); F_A—frequency of this allele in PsO cases; F_U—frequency of this allele in controls; CHISQ—basic allelic test chi-square; *p*—*p*-value; *p* adj—Bonferroni single-step adjusted *p*-values; OR—estimated odds ratio; SE—standard error; L95—lower bound of 95% confidence interval for odds ratio; U95—upper bound of 95% confidence interval for odds ratio.

**Table 2:** Comparison of the frequency of SNPs in the group of patients with type I PsO and the control group.

CHR	SNP	A1	F_A	F_U	CHISQ	*p*	*p* adj	OR	SE	L95	U95	Overlapped Gene	Annotation
6	rs3093662	G	0.129	0.0515	34.13	**5.15 × 10^−9^**	1.51 × 10^−6^	**2.73**	0.18	1.92	3.88	*TNF*	intronic
1	rs2201584	T	0.228	0.1396	18.36	**1.83 × 10^−5^**	0.00539	**1.82**	0.14	1.38	2.40	*IL12RB2*	intronic
5	rs3213094	A	0.105	0.1989	15.84	**6.90 × 10^−5^**	0.02029	**0.47**	0.19	0.33	0.69	*IL12B*	intronic
5	rs3212220	T	0.105	0.1989	15.84	**6.90 × 10^−5^**	0.02029	**0.47**	0.19	0.33	0.69	*IL12B*	intronic
1	rs6693065	G	0.289	0.2024	13.25	**0.0002732**	0.08032	**1.60**	0.13	1.24	2.07	*IL12RB2*	intronic
1	rs12142823	T	0.196	0.1237	13.14	**0.0002895**	0.08512	**1.73**	0.15	1.28	2.34	*IL12RB2*	5utr, 5upstream
5	rs1295686	A	0.170	0.2505	9.92	**0.001633**	0.48	**0.61**	0.16	0.45	0.83	*IL13/TH2LCRR*	intronic, non-coding intronic
5	rs20541	T	0.170	0.2486	9.50	**0.002059**	0.6052	**0.62**	0.16	0.46	0.84	*IL13/TH2LCRR*	coding nonsyn, non-coding
3	rs4135280	C	0.027	0.0643	6.64	**0.00997**	1	**0.41**	0.36	0.20	0.83	*PPARG*	intronic, 3downstream
10	rs1044268	A	0.119	0.1756	6.37	**0.01159**	1	**0.63**	0.18	0.44	0.91	*NRP1*	3utr
10	rs4582902	T	0.545	0.4715	6.08	**0.01367**	1	**1.34**	0.12	1.06	1.69	*ENTPD1*	intronic, non-coding intronic
22	rs2241042	A	0.329	0.4003	6.08	**0.01367**	1	**0.73**	0.13	0.57	0.94	*IL17RA*	intronic, non-coding intronic
21	rs2268288	C	0.151	0.2096	5.99	**0.01442**	1	**0.67**	0.17	0.48	0.93	*RUNX1*	intronic, 5upstream
10	rs734187	T	0.228	0.2901	5.40	**0.02011**	1	**0.72**	0.14	0.55	0.95	*NRP1*	intronic, 3downstream
21	rs2834655	A	0.252	0.3121	4.89	**0.02707**	1	**0.74**	0.14	0.57	0.97	*RUNX1*	intronic
21	rs2268278	A	0.286	0.3472	4.78	**0.02876**	1	**0.75**	0.13	0.58	0.97	*RUNX1*	intronic
11	rs2228055	G	0.024	0.0519	4.65	**0.03101**	1	**0.45**	0.38	0.21	0.95	*IL10RA*	coding nonsyn, 5upstream, 3utr, non-coding, 3downstream
10	rs11188484	A	0.384	0.3257	4.47	**0.03447**	1	**1.29**	0.12	1.02	1.64	*ENTPD1*	intronic, non-coding intronic
2	rs10515944	A	0.188	0.1442	4.46	**0.03474**	1	**1.38**	0.15	1.02	1.86	*CD28*	intronic
1	rs2064689	A	0.344	0.2872	4.43	**0.0353**	1	**1.30**	0.12	1.02	1.66	*IL23R*	intronic
1	rs10789224	C	0.340	0.2850	4.27	**0.0389**	1	**1.29**	0.12	1.01	1.65	*IL23R*	intronic
1	rs10489630	G	0.439	0.3802	4.16	**0.04142**	1	**1.27**	0.12	1.01	1.61	*IL23R*	intronic
9	rs10481593	A	0.180	0.2303	4.05	**0.04411**	1	**0.74**	0.15	0.54	0.99	*CD274*	intronic, non-coding intronic
9	rs1411262	A	0.245	0.2981	3.88	**0.04875**	1	**0.76**	0.14	0.58	1.00	*CD274*	intronic, non-coding intronic
11	rs2256111	G	0.537	0.4795	3.84	**0.04993**	1	**1.26**	0.12	1.00	1.59	*IL10RA*	coding syn, 5upstream, coding nonsyn, non-coding, 3utr

CHR—chromosome; SNP—single nucleotide polymorphism; A1—minor allele name (based on whole sample); F_A—frequency of this allele in PsO cases; F_U—frequency of this allele in controls; CHISQ—basic allelic test chi-square; *p*—*p*-value; *p* adj—Bonferroni single-step adjusted *p*-values; OR—estimated odds ratio; SE—standard error; L95—lower bound of 95% confidence interval for odds ratio; U95—upper bound of 95% confidence interval for odds ratio.

**Table 3:** Comparison of the frequency of SNPs in the group of patients with type II PsO and the control group.

CHR	SNP	A1	F_A	F_U	CHISQ	*p*	*p* adj	OR	SE	L95	U95	Overlapped Gene	Annotation
17	rs8069645	G	0.12	0.27	9.56	**0.00199**	0.5857	**0.38**	0.32	0.20	0.72	*STAT3*	intronic
8	rs7820268	T	0.23	0.36	6.38	**0.01154**	1	**0.54**	0.25	0.33	0.88	*IDO1*	intronic, 3downstream, non-coding intronic
1	rs7553796	A	0.61	0.48	6.37	**0.01163**	1	**1.72**	0.22	1.12	2.63	*IL6R*	intronic
1	rs4845618	G	0.62	0.49	6.02	**0.01414**	1	**1.70**	0.22	1.11	2.60	*IL6R*	intronic, 5upstream
17	rs9900213	T	0.06	0.15	5.88	**0.01531**	1	**0.34**	0.46	0.14	0.85	*STAT5B*	intronic, non-coding intronic
17	rs3816769	C	0.21	0.32	5.27	**0.02165**	1	**0.56**	0.26	0.33	0.92	*STAT3*	intronic, non-coding intronic, 3downstream
1	rs4845625	T	0.61	0.49	5.16	**0.02314**	1	**1.63**	0.22	1.07	2.49	*IL6R*	intronic, non-coding intronic
1	rs1041937	A	0.18	0.28	4.51	**0.03376**	1	**0.56**	0.28	0.33	0.96	*LGALS8*	intronic, coding nonsyn, non-coding, coding syn, 5upstream, 3downstream
1	rs3819001	G	0.09	0.04	4.46	**0.03480**	1	**2.16**	0.37	1.04	4.49	*TNFRSF18*	3utr, 3downstream
17	rs1026916	A	0.22	0.33	4.33	**0.03750**	1	**0.59**	0.25	0.36	0.98	*STAT3*	intronic
17	rs1053005	G	0.10	0.18	4.23	**0.03978**	1	**0.49**	0.35	0.25	0.98	*STAT3*	3utr, 3downstream
11	rs2228055	G	0.10	0.05	4.17	**0.04115**	1	**2.03**	0.35	1.01	4.06	*IL10RA*	coding nonsyn, 5upstream, 3utr, non-coding, 3downstream
17	rs2293154	A	0.09	0.17	4.14	**0.04190**	1	**0.48**	0.37	0.23	0.99	*STAT5A*	intronic, non-coding intronic, 3downstream
5	rs10940495	G	0.34	0.25	4.10	**0.04294**	1	**1.57**	0.22	1.01	2.42	*IL6ST*	intronic, non-coding intronic
10	rs10490938	A	0.28	0.19	4.03	**0.04462**	1	**1.60**	0.24	1.01	2.55	*NRP1*	intronic
2	rs231779	T	0.51	0.41	3.98	**0.04601**	1	**1.52**	0.21	1.00	2.30	*CTLA4*	intronic, 5upstream, non-coding intronic
17	rs744166	C	0.28	0.38	3.90	**0.04819**	1	**0.63**	0.24	0.40	1.00	*STAT3*	intronic

CHR—chromosome; SNP—single nucleotide polymorphism; A1—minor allele name (based on whole sample); F_A—frequency of this allele in PsO cases; F_U—frequency of this allele in controls; CHISQ—basic allelic test chi-square; *p*—*p*-value; *p* adj—Bonferroni single-step adjusted *p*-values; OR—estimated odds ratio; SE—standard error; L95—lower bound of 95% confidence interval for odds ratio; U95—upper bound of 95% confidence interval for odds ratio.

**Table 4:** The occurrence of genotypes and alleles for selected gene polymorphisms in PsO patients and the control group.

Genotypes and Alleles	Control N = 173	Psoriasis N = 150	OR (95% CI)	*p*-Value
***IL4*-590** **rs2243250**				
**CC**	115 66.5%	64 42.7%	**0.38 (0.24–0.59)**	**<0.0001**
**CT**	51 29.5%	77 51.3%	**2.52 (1.60–3.99)**	**0.0001**
**TT**	7 4.0%	9 6.0%	1.51 (0.55–4.17)	0.42
**C**	281 81.2%	205 68.3%	**0.50 (0.35–0.72)**	**0.0002**
**T**	65 18.8%	95 31.7%	**2.00 (1.39–2.88)**	**0.0002**
***IL6*-174** **rs1800795**				
**GG**	30 15.3%	31 20.7%	1.24 (0.71–2.17)	0.45
**GC**	107 63.2%	82 54.7%	0.74 (0.48–1.16)	0.19
**CC**	36 21.5%	37 24.6%	1.25 (0.74–2.10)	0.41
**G**	167 48.3%	144 48.0%	0.99 (0.73–1.35)	0.95
**C**	179 51.7%	156 52.0%	1.01 (0.74–1.38)	0.95
***IL10*-1082** **rs1800896**				
**GG**	47 27.2%	26 17.3%	**0.56 (0.33–0.96)**	**0.04**
**GA**	90 52.0%	81 54.0%	1.08 (0.70–1.68)	0.72
**AA**	36 20.8%	43 28.7%	1.53 (0.92–2.55)	0.10
**G**	184 53.2%	133 44.3%	**0.70 (0.51–0.96)**	**0.02**
**A**	162 46.8%	167 55.7%	**1.43 (1.05–1.95)**	**0.02**
***TGFB1* codon10** **rs1982073**				
**TT**	71 41.0%	119 79.3%	**5.50 (3.35–9.07)**	**<0.0001**
**TC**	56 32.4%	19 12.7%	**0.30 (0.17–0.54)**	**0.0001**
**CC**	46 26.6%	12 8.0%	**0.24 (0.12–0.47)**	**<0.0001**
**T**	198 57.2%	257 85.7%	**4.47 (3.03–6.58)**	**<0.0001**
**C**	148 42.3%	43 14.3%	**0.22 (0.15–0.33)**	***p* < 0.0001**
***CTLA4*-318** **rs5742909**				
**CC**	140 80.9%	110 73.3%	0.65 (0.38–1.09)	0.11
**CT**	32 18.5%	40 26.7%	1.60 (0.95–2.72)	0.08
**TT**	1 0.6%	0 0.0%	0.38 (0.02–9.45)	0.56
**C**	312 90.2%	260 86.7%	0.71 (0.44–1.15)	0.16
**T**	34 9.8%	40 13.3%	1.41 (0.87–2.30)	0.16
***STAT3*** **rs2293152**				
**GG**	73 42.8%	57 38.0%	0.84 (0.54–1.31)	0.44
**GC**	86 49.7%	89 59.3%	1.48 (0.95–2.30)	0.08
**CC**	14 7.5%	4 2.7%	**0.31 (0.10–0.97)**	**0.04**
**G**	232 67.1%	203 67.7%	1.03 (0.74–1.43)	0.87
**C**	114 32.9%	97 32.3%	0.97 (0.70–1.35)	0.87
***STAT3*** **rs4796793**				
**CC**	88 50.9%	87 58.0%	1.33 (0.85–2.07)	0.20
**CG**	69 39.9%	51 34.0%	0.78 (0.49–1.22)	0.28
**GG**	16 9.2%	12 8.0%	0.85 (0.39–1.87)	0.69
**C**	245 70.8%	225 75.0%	1.24 (0.87–1.75)	0.23
**G**	101 29.2%	75 25.0%	0.81 (0.57–1.15)	0.23
***FOXP3*** **rs2232365**				
**AA**	61 35.3%	63 42.0%	1.33 (0.85–2.08)	0.21
**AG**	40 23.1%	29 19.3%	0.80 (0.47–1.36)	0.41
**GG**	72 41.6%	58 38.7%	0.88 (0.57–1.38)	0.59
**A**	162 46.8%	155 51.7%	1.21 (0.89–1.65)	0.22
**G**	184 53.2%	145 48.3%	0.82 (0.60–1.12)	0.22

OR—odds ratio, CI—confidence interval.

**Table 5:** Primer sequences used in the study.

Gene Name	Forward Primer (5′–3′)	Reverse Primer (5′–3′)
*TNF*	CCAGGGACCTCTCTCTAATCA	TCAGCTTGAGGGTTTGCTAC
*IL1A*	CTGAAGAAGAGACGGTTGAGTT	GCTGACCTAGGCTTGATGATT
*FOXP3*	CTGCTCGCACAGATTACTT	GCAGCTTTGAGGTTGTTTG
*IL2*	AAGAAGGCCACAGAACTGAAA	GTCCCTGGGTCTTAAGTGAAAG
*CTLA4*	TTTTTCTTCTCTTCATCCCTGTCTT	CACACACAAAGCTGGCGAT

## Data Availability

The data that support the findings of this study are available from the corresponding author upon reasonable request.

## Supplementary Materials

The supporting information can be downloaded at: https://www.mdpi.com/article/10.3390/ijms24076061/s1.

## Abbreviations

CD28	Cluster of Differentiation 28
CD274	Cluster of Differentiation 274
CTLA4	Cytotoxic T cell antigen 4
ENTPD1	Ectonucleoside Triphosphate Diphosphohydrolase 1
FOXP3	Forkhead box P3
IDO1	Indoleamine 2,3-Dioxygenase 1
IL1A	Interleukin 1 alpha
IL2	Interleukin 2
IL4	Interleukin 4
IL6R	Interleukin 6 Receptor
IL6ST	Interleukin 6 Cytokine Family Signal Transducer
IL10RA	Interleukin 10 Receptor Subunit Alpha
IL12B	Interleukin 12B
IL12RB2	Interleukin 12 Receptor Subunit Beta 2
IL13	Interleukin 13
IL17RA	Interleukin 17 Receptor A
IL23R	Interleukin 23 Receptor
LGALS8	Galectin 8
NRP1	Neuropilin 1
PPARG	Peroxisome Proliferator Activated Receptor Gamma
RUNX1	Runt-related Transcription Factor 1
STAT3	Signal Transducer and Activator of Transcription 3
STAT5A	Signal Transducer and Activator of Transcription 5A
STAT5B	Signal Transducer and Activator of Transcription 5B
TGF-β1	Transforming Growth Factor β1
TNF	Tumor Necrosis Factor
TNFRSF18	Tumor Necrosis Factor Receptor Superfamily Member 18
